# Appendicite aigue sur hernie de Claudius Amyand chez un nouveau-né dans un tableau d’occlusion neonatale

**DOI:** 10.11604/pamj.2018.29.96.11357

**Published:** 2018-01-31

**Authors:** Hind Cherrabi, Salahoudine Idrissa, Hind Abouljaoud, Abdoulaye Harouna diallo, Karima Atarraf, Aziz El Madi, Khalid Khattala, Youssef Bouabdallah

**Affiliations:** 1 Chirurgie Pédiatrique, Hôpital Mère Enfant, CHU Hassan II Université Sidi Mohammed Ben Abdellah Fès, Maroc

**Keywords:** Hernie de amyand, appendicite intra herniaire, nouveau-né, Amyand's hernia, intrahernial appendicitis, newborn

## Abstract

La hernie de Claudius Amyand est définie par l'incarcération de l'appendice vermiculaire à travers le sac herniaire. La première appendicectomie fut réalisée en 1735. C'est une pathologie très rare chez l'enfant. De ce fait; la fréquence de cette pathologie est non encore établie. Nous rapportons l'observation d'un nouveau-né de 22 jours porteur d'une hernie inguino scrotale simple non suivi admis aux urgences dans un tableau de syndrome occlusif fait d'arrêt des matières et des gaz avec tuméfaction inguino-scrotale d'allure inflammatoire et des vomissements bilieux installés sur 2 jours. La prise en charge a consisté a une mise en condition et un bilan pré-anesthésique. L'exploration per opératoire a mis en évidence un appendice boudiné nécrosé dans sa moitié distale avec présence de fausses membranes. Le geste a comporté une appendicectomie et la fermeture du sac herniaire. L'évolution a été marquée par une reprise de transit 24h de post opératoire.

## Abstract (en)

Claudius Amyand's hernia is defined as the incarceration of the vermiform appendix in the hernia sac. The first reported appendicectomy was performed in 1735. It is very rare among children, hence its frequency is not yet established. We report the case of a 22-day old new born with untreated simple inguinoscrotal hernia who had an urgent admission to the Emergency Department due to occlusive syndrome with no evacuation of faeces and no passing of flatus, inflammatory inguinoscrotal swelling and bilious vomiting persisting for 2 days. Treatment was based on the restoration to good health and on preanesthetic assessment. Intraoperative exploration showed necrotic bulging appendix in its distal half with false membranes. Appendectomy was performed with closure of the hernia sac. Patient's evolution was marked by resumption of intestinal transit 24h after surgery.

## Introduction

La hernie de Claudius Amyand est caractérisée par la présence de l'appendice vermiculaire dans la hernie inguinale, que l'appendice soit inflammatoire ou non. Elle a été décrite pour la 1^ère^ fois par Claudius Amyand en 1735 a l'Hôpital Saint George a Londres chez un enfant de 11 ans qui fut admis pour hernie inguinale droite compliquée de fistule stercorale scrotale droite. Amyand à découvert à l'exploration par une incision inguinale droite un épingle au sein du stercolithe. Il procéda à une appendicectomie avec résection fermeture du sac herniaire et mise à plat de la fistule. Les suites opératoires furent simples.

## Patient et observation

**Figure 1:**
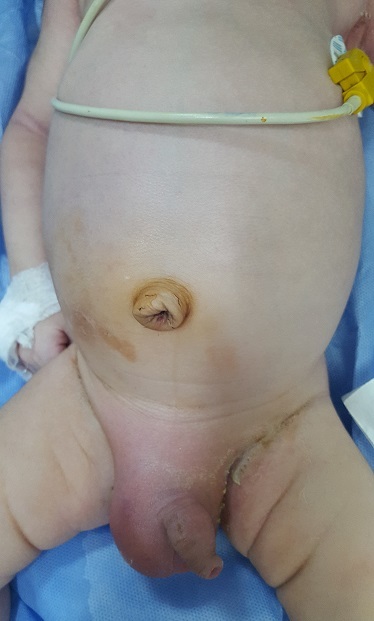
Aspect préopératoire de la hernie inguino scrotale ‘

**Figure 2:**
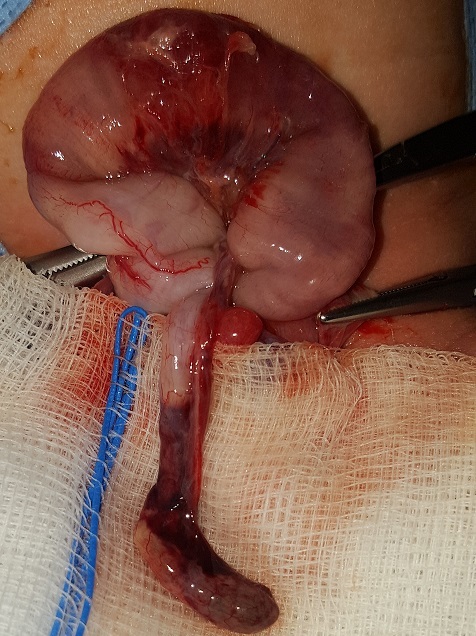
Aspect peropératoire de l’appendicite intra herniaire avec des fausses membranes

Ce nouveau né de 22 jours de sexe masculin a été transféré pour la prise en charge d'occlusion néonatale fébrile évoluant depuis 2 jours. A l'admission le nouveau-né était conscient, rose, hypotonique, fébricule à 37,7°C et stable sur le plan hémodynamique et respiratoire. L'examen abdominal a objectivé un abdomen légèrement distendu avec une tuméfaction inguino-scrotale droite douloureuse d'allure inflammatoire. L'épreuve de transillumination est négative a droite. Le testicule gauche en place (Figure 1). Après une mise condition ayant consisté à mettre le nouveau né dans une table chauffante, sous diète et mise en place d'une sonde gastrique de bon calibre, une voie veineuse périphérique permettant d'administrer une réhydratation, une ration de base adaptée, une triple antibiothérapie intra veineuse associant C3G, aminosides et métronidazole ainsi qu'un traitement antalgique. Le bilan biologique pré opératoire a montré une Hémoglobine = 12g/dl, une fonction rénale correcte avec une hyponatrémie à 129mmol/l. Le bilan radiologique représenté par une radiographie thoraco-abdominale en position debout a révélé des niveaux hydro aréiques grêliques et coliques. Au terme du bilan clinique et radiologique le diagnostic d'une hernie inguino scrotale droite compliquée d'occlusion néonatale a été retenu. Le nouveau né a été admis au bloc opératoire après 6h de réanimation hydro-éléctrolytique, installé en décubitus dorsal sur table chauffante. On a procédé à une incision du pli abdominal inférieur droit, après passage à travers le fascia transversalis, le sac herniaire à contenu digestif a été repéré avec dissection minutieuse tout autour. A son ouverture la portion distale de l'appendice (Figure 2) à aspect nécrosé a été mise en évidence avec incarcération d'un segment du colon ascendant et présence de fausses membranes en regard. Le reste de l'exploration des anses a objectivé leur aspect viable. On a noté un défaut d'accollement du colon droit. Le geste chirurgical a consisté à une appendicectomie avec réintégration du colon hernié et résection puis fermeture du sac herniaire (Figure 2). Les suites post opératoires ont été marquées par la reprise du transit après 24h avec affaissement abdominal et disparition des vomissements d'où la décision de sa sortie.

## Discussion

La hernie de « Claudius Amyand » est une entité très rare chez l'enfant. La présentation clinique est le plus souvent celle d'une hernie étranglée compliquée ou non d'un syndrome occlusif fébrile [1-3]. Le bilan biologique est radiologique ont pour objectifs respectivement d'évaluer le retentissement hydro électrolytique et de rechercher les complications éventuelles. Une prise en charge rapide et adaptée s'impose par la mise en condition et le geste chirurgical doit être précoce après une bonne préparation de l'enfant. Le diagnostic positif est le plus souvent fait en per opératoire à la constatation d'un appendice vermiculaire situé au sein du sac herniaire qu'il soit inflammé ou non. L'intervention consiste à une appendicectomie et résection fermeture du sac herniaire [4-7]. Sur une série turque récente publiée en 2009 de 1090 enfants porteurs d'une hernie inguinale, 33 de ces dernières étaient incarcérées et 12 patients présentaient une hernie de Claudius Amyand. Il s'agissait toujours de garçons avec une médiane d'âge moyen de 40 jours (extrême qu'un jour 14 mois) [8, 9]. Parmi les 12 hernies de Claudius Amyand, deux appendices étaient normaux six étaient inflammatoires et 2 étaient le siège d'inflammation séreuse de signification incertaine, mécanique ou infectieuse. Plusieurs cas hernie de Claudius Amyand du côté gauche ont été rapportés.

## Conclusion

L'issue de l'appendice à travers le sac herniaire définit la hernie de Claudius Amyand. La 1^ere^ description de cette entité a été faite par Amyand qui a réalisé une appendicectomie avec résection fermeture du sac herniaire. Les séries de la littérature rapportant des cas surtout chez les adultes. Chez l'enfant; cette pathologie demeure si rare et le diagnostic est posé le plus souvent dans un tableau de hernie étranglée à l'exploration per opératoire. La prise en charge passe par une mise en condition adaptée et le traitement chirurgical doit etre le plutôt possible. Le geste consiste à une appendicectomie avec resection fermeture du sac herniaire.

## Conflits d’intérêts

Les auteurs ne déclarent aucun conflit d'intérêts.
